# On the Orientation Error of IMU: Investigating Static and Dynamic Accuracy Targeting Human Motion

**DOI:** 10.1371/journal.pone.0161940

**Published:** 2016-09-09

**Authors:** Luca Ricci, Fabrizio Taffoni, Domenico Formica

**Affiliations:** Unit of Biomedical Robotics and Biomicrosystems, Department of Engineering, Università Campus Bio-Medico di Roma, via Àlvaro del Portillo 21, 00128 Rome, Italy; Universitat Zurich, SWITZERLAND

## Abstract

The accuracy in orientation tracking attainable by using inertial measurement units (IMU) when measuring human motion is still an open issue. This study presents a systematic quantification of the accuracy under static conditions and typical human dynamics, simulated by means of a robotic arm. Two sensor fusion algorithms, selected from the classes of the stochastic and complementary methods, are considered. The proposed protocol implements controlled and repeatable experimental conditions and validates accuracy for an extensive set of dynamic movements, that differ in frequency and amplitude of the movement. We found that dynamic performance of the tracking is only slightly dependent on the sensor fusion algorithm. Instead, it is dependent on the amplitude and frequency of the movement and a major contribution to the error derives from the orientation of the rotation axis w.r.t. the gravity vector. Absolute and relative errors upper bounds are found respectively in the range [0.7° ÷ 8.2°] and [1.0° ÷ 10.3°]. Alongside dynamic, static accuracy is thoroughly investigated, also with an emphasis on convergence behavior of the different algorithms. Reported results emphasize critical issues associated with the use of this technology and provide a baseline level of performance for the human motion related application.

## Introduction

Human kinematic tracking by means of wearable IMU sensors that are directly attached to the body is emerging as a promising alternative to stereophotogrammetry based systems (ranked as the gold standard). An IMU comprises tri–axial accelerometers and gyroscopes and is typically coupled with a magnetic flux sensor. Sensor fusion of the IMU readings allow measuring 3D orientation with respect to a fixed system of coordinates [1]. Therefore, when an IMU is firmly attached to a human body segment, it is possible to obtain an estimate of its absolute orientation. Furthermore, when multiple IMUs are attached to different body segments their relative orientation can be combined to measure human motion [2]. Extensive research effort within this specific field of application has been committed to: the investigation of new sensor fusion algorithms for orientation estimation [3–6]; the definition of protocols for practical usage of wearable IMUs with humans [7–9]; the extension to different scenarios [10–12]. Surprisingly, less attention has been dedicated to rigorously assessing the accuracy in estimating orientation attainable with a wearable IMU during typical motion conditions. Technical specification of commercial systems reported by vendors are presented with caveats and are poorly documented, e.g. the dynamic accuracy is reported without detailing the testing setup and amplitude and bandwidth of testing movements. The main contribution of this work is in providing a systematic characterization of the accuracy in orientation measuring under controlled and repeatable conditions and using state–of–the–art sensor fusion algorithms. The latter are selected within the classes of Kalman filters (KF) and Complementary filters (CF) to which most of developed methods pertain. The assessment protocol we present comprises evaluation of both absolute (single IMU compared to reference) and relative (pairwise comparison between IMUs measurements) accuracy in static and dynamic scenarios. To the best of our knowledge, this is the first study that investigates accuracy in orientation tracking of IMU devices in such a detail, providing baseline data valuable for human motion capture researchers. The paper is organized as follows: first, we describe the experimental setup, define our protocol and the data analysis. Then, we report obtained results for each trial and, finally, we conclude with a critical discussion of the findings. Beforehand, to put our contribution in perspective, next paragraph provides a brief review of the relevant literature on the topic.

### Related work

Existing studies in the literature investigating the accuracy of orientation measurement with an IMU consider static and dynamic validation mostly under manually generated conditions. In [13], Cutti et al. tested 4 IMUs rigidly attached to a manually rotated plank. Mean angular velocities of 180°/*s* and 360°/*s* were generated with the help of a metronome and a worst case angular error for the two velocities was found to be 5.4° and 11.6° respectively. The oscillatory motion of a pendulum had also been considered for the dynamic accuracy assessment, e.g. in [14] and in [15]. In both studies, an optical system was used as reference. In the first one, a worst case RMS error was found to be in the range 8.5° ÷ 11.7° for the IMU factory orientation estimator (a KF) and much lower 0.8° ÷ 1.3° for the algorithm developed by the authors. In the latter, the mean RMS error range was in between 1.9° and 3.5°. Differently, Picerno et al. [16] focused on consistency in orientation measurement of multiple IMUs by presenting a spot check for device assessment. They pointed out critical limitations in measuring relative orientation with IMUs, reporting errors as large as 11.4°. Presently, the only example of accuracy evaluation under controlled conditions, i.e. with an experimental setup and protocol capable of providing well defined and repeatable testing movements is that of [17, 18]. They compared different commercial IMU systems for motion tracking against an optical system and used an instrumented gimbal table to generate static and dynamic motion conditions. In [17], their focus was on the effect of angular velocity magnitude on orientation error and reported results suggest a significant effect of this variable for all tested devices. Mean errors w.r.t optical reference for the case of 90°/*s* and 180°/*s* angular velocities were found to be around 3° and 7°. In [18], they used the same setup to analyse the effect of time on accuracy reporting a significant decrease in accuracy over subsequent motion trials. A recent paper from Bergamini et al. [19] investigated the accuracy of different sensor fusion approaches for orientation estimation with IMUs during several manual and locomotion tasks. For their setup the reported mean errors varied greatly both depending on the task and the type of rotation (heading or attitude) and ranging from about 5°, for manual tasks, up to 21°, for locomotion.

## Materials and Methods

### Experimental setup

**Fig 1:**
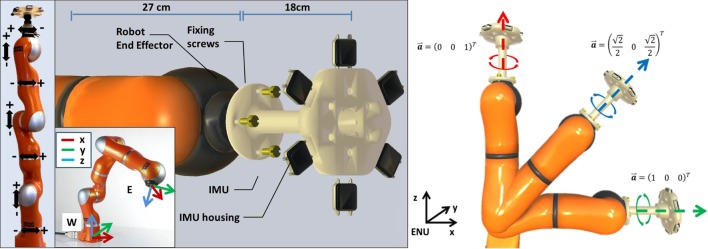
The figure illustrates the experimental setup used for performing the accuracy assessment. From the left to the right the figure display: a view of the robotic arm, where all the 7 DoF axes are indicated; the definition of the world (W) and the end-effector (E) coordinate frames of the robot; the custom tool devised with the fixation mechanism used to rigidly attach the 6 IMUs to the robotic system; the 3 different configuration of the robotic arm used in the experimental protocol.

The experimental setup for this study is shown in Fig 1 and consists a commercial set of IMUs and a robotic arm. The IMUs are manufactured by APDM Inc. and are 6 wearable sensor units in total (“Opal” type). They provide real time inertial measurements and orientation in a North-West-Up (NWU) frame computed via an embedded KF algorithm. Synchronized measurements from the units can be retrieved up to a maximum rate of 128 Hz. The robotic arm is the lightweight manipulator LWR 4+ manufactured by KUKA GmbH. It features 7 rotational joints distributed along an anthropomorphic kinematic chain and a high repeatability ±0.05 mm (ISO 9238). Depending on the specific joint, angular velocities from a minimum of ±110°/*s* to a maximum of ±240°/*s* can be generated. Relative orientation of the robot joints is measured by absolute 16-bit magnetic encoder corresponding to an accuracy in joint orientation < 0.01°. Joint odometry and pose of the end-effector (EE) can be retrieved from the robot up to a maximum rate of 1 kHz. A connection tool, shown in Fig 1, was designed to allow the mechanical linkage of the 6 IMUs to the robotic arm EE. The overall weight of the tool and the 6 units is about 1.5 Kg, a value below the maximum payload of the robot (14 Kg). The whole system was connected to a laptop for data collection, respectively via an Ethernet connection for the robot and a dedicated wireless connection for the IMUs.

### Experimental protocol

The experimental protocol we designed consists of a dynamic and a static validation. The dynamic validation tests IMUs performance during sinusoidal rotation generated along the following axes:

1. $\vec{\text{a}}={\left[1\;0\;0\right]}^{T}$
2. $\vec{\text{a}}={\left[\sqrt{2}/2\;0\;\sqrt{2}/2\right]}^{T}$
3. $\vec{\text{a}}={\left[0\;0\;1\right]}^{T}$

defined w.r.t. an East-North-Up (ENU) system of coordinates as shown in Fig 1. For each configuration or the robotic arm, a set of sinusoidal movements was generated using a single or a pair of aligned robot joints.

The sinusoidal rotation was varied across 7 different frequencies and 6 different amplitudes. The frequencies were selected in order to provide good coverage in the bandwidth characterizing the majority of human movements: 75% of the spectral energy is below 5 Hz [20–22], and mostly concentrated around 1 Hz, e.g. for common activities of daily living (ADL). They were chosen by uniformly sampling in a logarithmic scale representation of the bandwidth of human motion and vary from a minimum of 0.18 Hz to a maximum of 5.6 Hz. The amplitude values were selected in accordance to a safety constraint on the maximum torque sustainable at the joint level by the robotic arm (with the given payload) and were generated by using a single joint {±3,±5,±9} or a pair of aligned and synchronously rotating joints {±6,±10,±18}.

Each dynamic movement was applied for 20 s and was followed by a rest period of 40 s, to ensure any transitory effect from former movement trial to be exhausted prior the execution of the next movement. The matrix of test in Table 1 was repeated for 3 different trials in which the orientation of the axis of rotation ($\vec{a}$) was varied as previously described. This allows to validate the IMU performance both separately for the attitude (configuration 1) and the heading tracking (configuration 3) and together for the attitude and heading case (configuration 2). In fact, the rotation vector is oriented: perpendicular (90.2 ± 0.97°) with respect to the gravity, parallel (0.9 ± 0.56°) to it and midway between the two conditions (44.7 ± 1.27°). Each trial was repeated for 5 times, generating 30 different datasets (5 repetitions × 6 IMUs) for each element in Table 1. The static performance part of the protocol comprises a pure static (PS) evaluation, in which the set of 6 IMUs is kept stationary for 1 h, and a static after motion (SaM) part, that evaluates the static behavior of the orientation soon after a dynamic movement is terminated. The purpose of the latter is to test the convergence time of the orientation: the orientation is evaluated over a period of 30 s, started at the termination of the dynamic movement trial. The convergence was evaluated with respect to the termination of the minimum and maximum dynamics movements, i.e. element (1, 1) and (6, 5) of the matrix in Table 1, for the 3 trials described above.

**Table 1:** Matrix of movement trials for dynamic accuracy validation expressed as (± amplitude in degrees, frequency in Hz): amplitudes are varied row–wise and frequencies are varied column-wise.

(±3, 0.18)	(±3, 0.32)	(±3, 0.56)	(±3, 1.00)	(±3, 1.78 Hz)	(±3, 3.16)	(±3, 5.62)
(±5, 0.18)	(±5, 0.32)	(±5, 0.56)	(±5, 1.00)	(±5, 1.78 Hz)	(±5, 3.16)	-
(±6, 0.18)	(±6, 0.32)	(±6, 0.56)	(±6, 1.00)	(±6, 1.78 Hz)	(±6, 3.16)	(±6, 5.62)
(±9, 0.18)	(±9, 0.32)	(±9, 0.56)	(±9, 1.00)	(±9, 1.78 Hz)	-	-
(±10, 0.18)	(±10, 0.32)	(±10, 0.56)	(±10, 1.00)	(±10, 1.78 Hz)	(±10, 3.16)	-
(±18, 0.18)	(±18, 0.32)	(±18, 0.56)	(±18, 1.00)	(±18, 1.78 Hz)	-	-

### Sensor fusion for orientation estimation

The algorithms we used for estimating the IMU’s orientation are selected from the non–linear CF [5, 6, 23, 24] and KF [3, 4] classes. For the CF class, we implemented in Matlab a recent algorithm that uses a Gauss–Newton algorithm (GNA) optimization, proposed in [6]. The tunable parameters were set according to recommendations by the authors: s^2^ and *ϵ*_*M*_ = 2 uT).

- filter gain *β* = 0.0756;
- dynamic acceleration threshold *ϵ*_*A*_ = 0.25 m/s^2^;
- magnetic perturbations threshold *ϵ*_*M*_ = 2 uT.

For the KF class, we used the embedded implementation available from our commercial system (firmware version 03/05/2014), which is conveniently tuned for human motion tracking and provided with reference values for accuracy [25]:

- static accuracy of 1.15° RMS (roll/pitch angles) and 1.50° RMS (yaw angle);
- dynamic accuracy of 2.8° RMS.

### Data analysis

The dataset resulting from a trial was made of a sequence of quaternions describing IMU body frame (B) orientation with respect to a global (G) North-West-Up frame, i.e. (^*GB*^***q***), and a companion sequence of reference quaternions from the robotic arm that express the orientation of the robot EE with respect to a world (W) frame fixed on its base segment, i.e. (^*WE*^***q***). Prior to perform the data analysis, the collected datasets were re-sampled at a constant rate of 128 Hz by using quaternion Spherical Linear intERPolation (SLERP). Data synchronization was achieved by fitting a linear model describing the clock difference between the two systems (clock offset and skew) on time difference measures obtained from correlations of the angular velocity *ℓ*^2^ norm, measured by the IMU and the robot, at different time periods. The orientation error metric on the quaternion space was defined as:

$$\Phi \left(q_{A},q_{B}\right)=2\;cos^{-1}\left(\left|q_{A}\cdot q_{B}\right|\right)$$ (1)

that is the length of the shortest path, i.e. a geodesic, connecting the two quaternions (***q***_*A*_,***q***_*B*_) on the 4–dimensional hypersphere where they are defined. In order to consistently evaluate accuracy with this metric, the IMU and the robot quaternion sequences were first referred to the same system of coordinates, i.e.:

$${}^{WG}q\;⊗{\;}^{GB}q\left(t\right){=}^{WE}q\left(t\right)\;⊗{\;}^{EB}q$$ (2)

where ⊗ is the quaternion multiplication symbol. The computation of the constant frame misalignment terms, ^*WG*^***q*** and ^*EB*^***q***, was performed with the method described in [26]. Then, two performance indexes of orientation tracking were extracted from the data: absolute and relative accuracy. The absolute accuracy (*ϵ*_*A*_) determines the capability of each IMU to correctly measure orientation against an absolute reference (i.e. the robot arm). It is evaluated as:

$$\epsilon_{A}=\Phi \left(q_{I_{i}},\;q_{GT}\right),\;i\;=\;1,\;\ldots ,\;6$$ (3)

where, ***q***_*I*_*i*__ is the measure from *i*–th IMU and ***q***_*GT*_ is the ground truth data. For each test on the matrix in Table 1, 30 datasets of absolute accuracy measures are obtained. The relative accuracy (*ϵ*_*R*_) accounts for differences in orientation measures from pairs of IMUs. Though being independent from the actual value of the orientation, it is a crucial quantity for all applications where relative motion is of interest, e.g. human joint angle measurement. It is computed as:

$$\epsilon_{R}=\Phi \left(q_{I_{i}},\;q_{I_{j}}\right),\;i\;\neq \;j$$ (4)

where, for each test in Table 1, all the possible combinations of IMU pairs generate 75 datasets of relative accuracy measurements.

## Results

The results throughout this section will be reported as the median, the inter quartile range (IQR) and the error upper bound (UB) defined using the 95^th^ quantile. This is motivated by the fact that, due to the characteristic of the metric on quaternion space defined in Eq (1) (e.g. it is always a positive quantity), the distribution of data is skewed and it is not possible to represent their statistics by using a finite set of parameters (e.g. the mean and the standard deviation for the Gaussian distribution case). In consideration of that, we used robust statistical measures, i.e the median and the quantiles, in order to perform our analysis.

### Static accuracy

**Fig 2:**
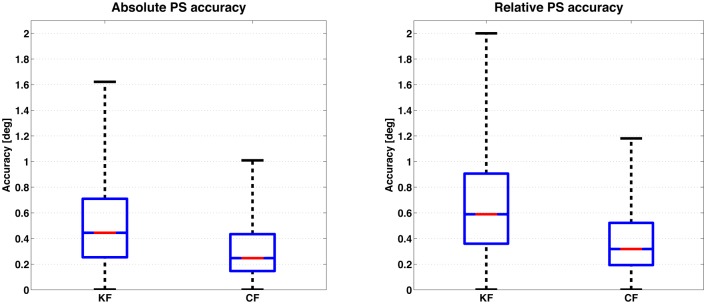
The boxplots represent absolute (left) and relative (right) accuracies for the *pure static*(PS) trial of static part of the protocol. The first and the second bar report respectively the data obtained by using the KF and the CF algorithm.

**Fig 3:**
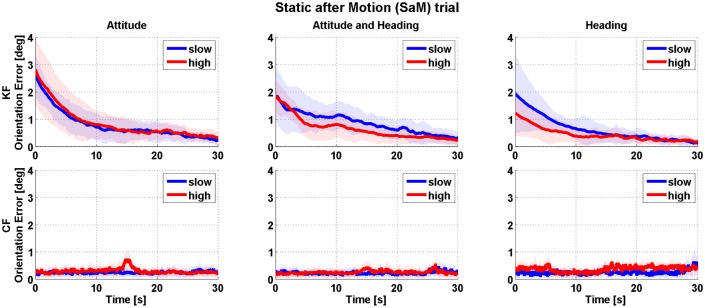
Results for the static after motion (SaM) trial: the solid line represents the median of the orientation error (i.e. the distance from the estimate at convergence) and its associated inter quartile range (colored shadow). Graphs are organized according to the type of trial, *heading*), the sensor fusion algorithm selected (*KF* or *CF*) and the type of dynamic movement considered (*slow* and *fast*).

The Fig 2 reports the results for the PS trial. Each bar represents the distribution of the cumulative error obtained from the IMUs under test, respectively for the case of the KF and the CF algorithm. Pertaining absolute accuracy, a median value of 0.44° (KF) and 0.25° (CF) was obtained and the maximum was found to be 1.62° (KF) and 1.01° (CF). For the relative accuracy, slightly higher median values were obtained, 0.58° (KF) and 0.32° (CF), while the maximum error was found to be 2.00° (KF) and 1.18° (CF). The IQR increased when passing from absolute to relative accuracy, respectively +20% for KF and +8% for the CF filter. Furthermore, the statistical significance of the results was investigated for a population using Wilcoxon rank–sum test, as the dataset was found not Gaussian distributed (Lilliefors test). To the purpose of the analysis, median values from each IMU dataset were considered. The filtering algorithm was found to have a significant effect on the results(*p* < 0.05), favoring the CF algorithm in the static case. The results from the SaM trial are reported in Fig 3. Each row in the graph is associated with a sensor fusion algorithm, the KF on the top and the CF on the bottom, while each column identifies one of the 3 trials, i.e. *attitude*, *attitude and heading* and *heading*). The plots show the trend in convergence of the orientation error from the end of a dynamic movement (at time 0 s) to a stable, static estimate. A neat difference in the convergence rate of the two sensor fusion algorithms used is observed: the KF algorithm takes 10 s to reach a stable estimate (within 1°) but independently of the type of trial and dynamics. Instead, the CF algorithm results in a noisier (due to the quantized optimization step), but stable convergence already at the beginning of the static stage.

### Dynamic accuracy

**Fig 4:**
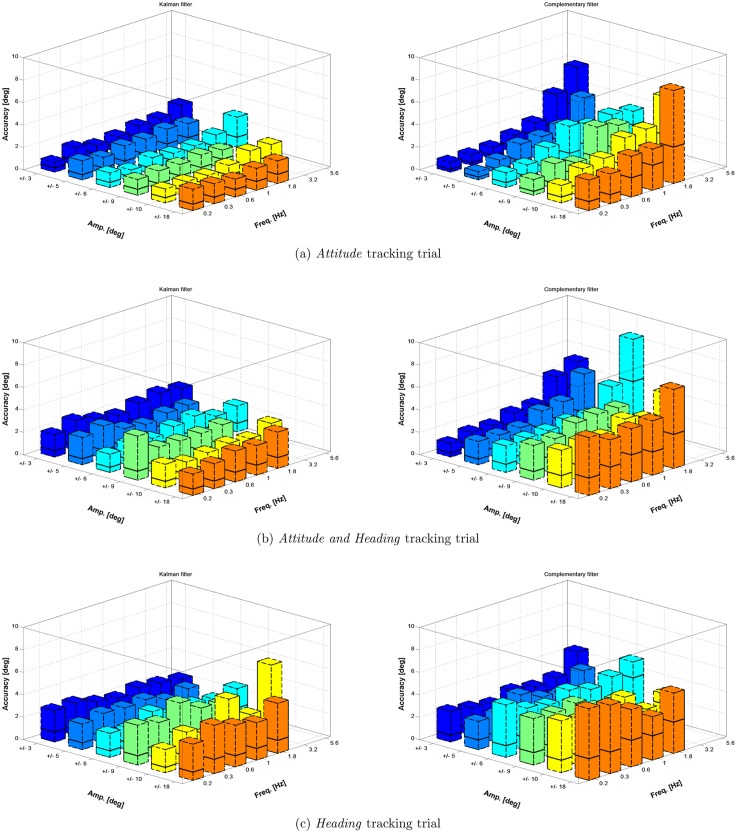
The figure reports the dynamic absolute accuracy absolute obtained with the KF algorithm (left column) and CF algorithm (right column). It is represented as the 95% error range (colored box) and the median value of results (solid black line).

**Fig 5:**
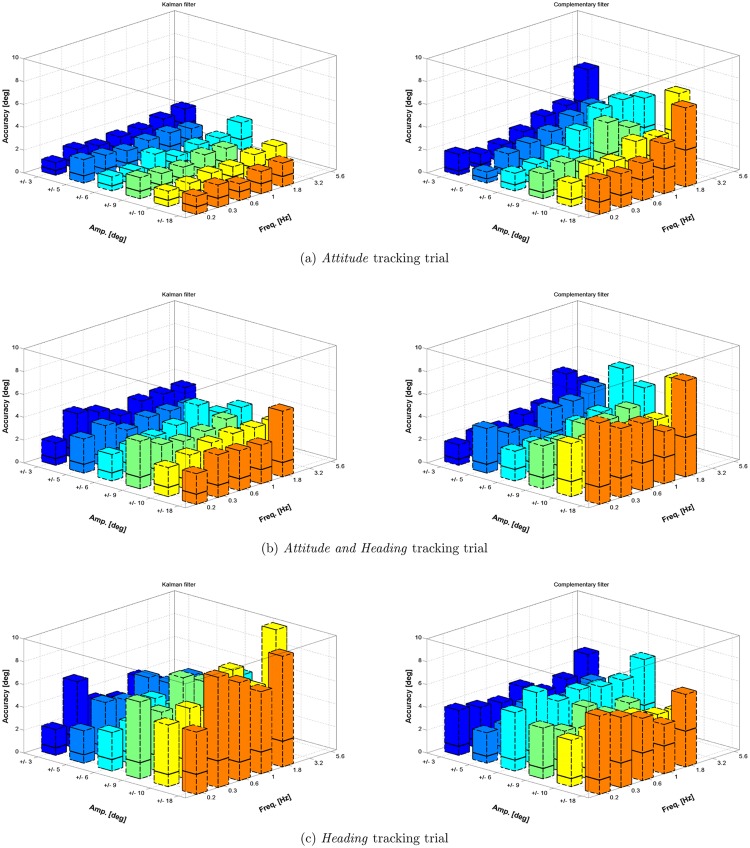
The figure reports the relative accuracy obtained with the KF algorithm (left column) and CF algorithm (right column). It is represented as the 95% error range (colored box) and the median value of results (solid black line).

The results from the dynamic validation protocol are reported in Fig 4 for the absolute accuracy and in Fig 5 for the relative accuracy. Each figure displays, on the left column, results for the KF algorithm and, on the right column, results for the CF algorithm. Each row represents one of the 3 tracking trials (i.e. attitude, attitude and heading, and heading), while x and y axes of each subplot represents the parameters (amplitude and frequency) of movements for each test in Table 1. Errors are represented as the median value (black line) and the range from 0 to the 95% UB (colored box). Each box is the cumulative distribution of the error from all the datasets pertaining the specific trial and element in the matrix of Table 1. Besides graphical trend representation, numerical values for the median and the 95% UB are provided as supplementary material in the S1 Table.

**Fig 6:**
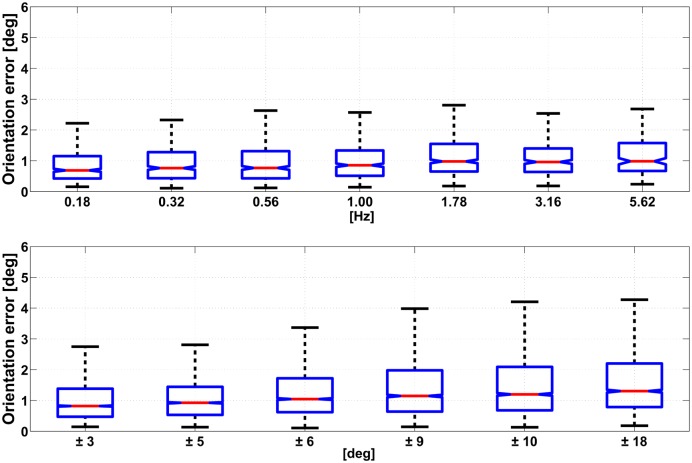
The figure reports the trend in the absolute orientation error computed with the KF, for varying movement frequency (top) and range of motion (bottom). Data are represented as the median value (red line), IQR (blue box) and minimum and maximum values (black whiskers). Outliers are removed from each dataset using a ±3 IQR threshold: respectively 55 and 53 datapoints out of 19440 from the top and the bottom graph.

**Fig 7:**
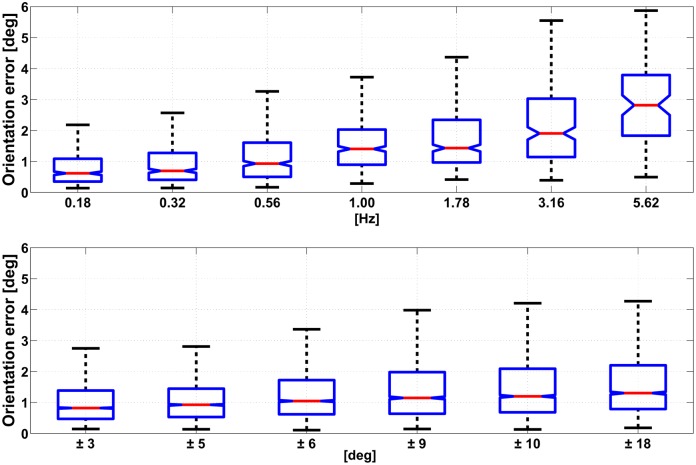
The figure reports the trend in the absolute orientation error computed with the CF, for varying movement frequency (top) and range of motion (bottom). Data are represented as the median value (red line), IQR (blue box) and minimum and maximum values (black whiskers). Outliers are removed from each dataset using a ±3 IQR threshold: respectively 16 and 32 datapoints out of 19440 from the top and the bottom graph.

A statistical analysis was performed to investigate the effect of the following variables:

1. type of sensor fusion algorithm (KF or CF);
2. type of accuracy metric (absolute or relative);
3. value of the frequency given the sensor fusion algorithm (7 and 7 groups);
4. value of the amplitude given the sensor fusion algorithm (6 and 6 groups);
5. type of trial (*attitude*, *heading* or *attitude and heading* trial);

The level of significance *α* was set to 0.05 for all statistical tests. The median value of the error during the 20 s movement trial was considered for the analysis. For each type of trial and element in the matrix Eq 1, a number of 30 and 75 samples were respectively available for absolute and relative error measurements. The statistical analysis was performed with the following procedure: first, the hypothesis of non Gaussianity was verified using a Lilliefors test. Then, for comparisons between 2 groups (variable set 1 and 2) we used a Wilcoxon rank–sum test. For the case in which multiple groups are compared (variable set 3, 4 and 5) a Kruskal–Wallis one–way ANOVA was considered, followed by post-hoc comparisons with Tukey–Kramer correction. Pertaining the type of sensor fusion algorithm variable, the KF algorithm performed slightly better than the CF with respect both to absolute accuracy (median value of 0.47° and 0.77° respectively) and to relative accuracy (median value of 0.48° and 0.83° respectively) with a statistical significance *p* < *α*. A significant effect was found for the type of accuracy metric variable: orientation errors from the IMU measurements were lower when an absolute accuracy metric was considered rather than a relative one. The statistical effect of the value of the movement frequency on the orientation error was found to be significant (*p* < *α*). From post-hoc analysis a number of sets were identified: elements within the same set had no significantly different effect on the orientation error while elements from different sets had. For the case of the KF algorithm those sets were: {0.18, 0.32, 0.56, 1.00} and {1.78, 3.16, 5.62}, where all the values are expressed in Hz. For the case of CF the following groups were identified: {0.18, 0.32},{0.56}, {1.00, 1.78, 3.16},{5.62}. The variation of the amplitude of the movement had a significant effect on the error (*p* < *α*). As for the above case of the frequency, the post-hoc analysis produced a number of different sets with significant effect on the accuracy. In this case those sets were the same for the KF and CF algorithm: {±3}, {±5}, {±6}, {±9,±10} and {±18}. In addition to that, the median error in orientation tends to raise with the increasing value of frequency and amplitude considered in the protocol. A visualization of this trend is displayed in Figs 6 and 7, respectively for the KF and CF algorithms. The type of trial variable, which corresponds to exciting the IMU with a different configuration of the robotic arm as shown in Fig 1, was found to impact significantly the orientation error. The median accuracies were 0.7°, 1.0° and 1.4°, respectively for *attitude*, *attitude and heading* and the *heading* case.

## Discussion

This paper presented a systematic study of IMU accuracy in measuring the orientation for a static and a range of dynamic movements with a bandwidth typically found in human motion scenarios. Considering that all the present studies provide discordant results, mainly obtained without well–controlled experimental conditions, the objective of the work was to provide the scientific community with a reliable baseline of values for the performance achievable with this technology. Existing literature on the topic has proposed 2 major approaches: the first employs either manually operated or motor controlled mechanical systems (e.g. motorized gimbal Table [17], pendulum [14, 15], Plexiglas plank) while the second carries out the assessment directly on the human subject [10, 19]. The first approach tends to highlight limitations inherent in the IMU by eliminating the sources of error that are due to the human factor (e.g. soft tissue artifacts or sensors misalignment). The second approach tends towards a more realistic validation scenario with respect to human related applications. The protocol we devised exploits the benefits of the first approach by using a robotic platform, which guarantees controlled and repeatable experimental conditions, while selecting dynamic sinusoidal movements in the bandwidth of human motion (in pursuance of the simulation of a realistic use case). The disturbances specifically induced by the robotic platform were quantified beforehand and found to be negligible (see S1 Fig in the appendix for further details). Instead, the local perturbation of the magnetic field induced by the environment was purposely not controlled during the experimentation. Despite this could have an impact on performance (specifically for the heading scenario), in a real use case scenario the homogeneity of the magnetic field can not be guaranteed. As in this study we focused on typical use case scenario performance, we leave the assessment of the performance under controlled magnetic field perturbation to a future research endeavor. The evaluation of static accuracy was performed for a PS and a SaM scenarios. The PS protocol, or slight variations to that, are often found in related literature, e.g. in [13–15] and partly in [17]. The main difference with our approach is in the test duration: 10 s in [13], 1 s in [14] and 30 s in [17]. Therefore, we are only partially provided with benchmark values from literature. Also, specifications from vendor are expressed as Euler angles and with no detail about the assessment procedure. Despite those considerations, the median errors we reported comply well with the accuracy requirement of human motion capturing for both sensor fusion techniques and, after proper conversion to Euler angles, result in smaller values than vendor’s specifications (KF case). Also, the maximum errors in static accuracy, that are directly affected by the adaptations of the sensor fusion algorithms during the trial (e.g. to track changes in environmental conditions) are within acceptable level for the human motion application (maximum error is 1.62°). Interestingly, the higher errors obtained for the relative accuracies in the same trial suggest that those adaptations are not necessarily consistent among the different IMUs and is in agreement with the discussion in [18]. Overall, our results show that a good stability of static orientation estimate with an IMU can be achieved over a period of 1 hour. The SaM protocol part of the static accuracy assessment investigated the time of convergence of the orientation estimate after the extinction of a dynamic movement. The major difference observed here is in the behavior of the two algorithms. The CF algorithm has an immediate convergence, driven by the GNA regression and it is immediately stable within a level of accuracy comparable to the PS trial. Instead, the KF requires about 10 s to reach a stable estimate, though independently from the previous movement’s dynamics or type of trial. This settling time is the result of small errors in the estimate of the bias components of the gyroscope that are accumulated during the dynamic movement and that are corrected when the sensor is still. This is not observed for the CF algorithm as gyroscopes’ biases are not estimated (and sensor data are then assumed pre–calibrated for biases). The dynamic accuracy assessment of orientation tracking comprised a set sinusoidal movements with varying frequency and amplitude. The assumption of periodic movements to be representative of human motion repertoire is not uncommon in the literature (e.g. in human gait) and has been used as a modeling assumption for sensor fusion algorithms [27]. The novelty of our protocol implies that an actual comparison with data from previous studies is not feasible. Also, dynamic accuracy specifications from vendors comes with no information about the assessment protocol. The only similar work in the literature is the one by Lebel and colleagues [17], where two different dynamic conditions are explored by varying a constant angular velocity movement from a value of 90°/s to 180°/s. Despite our protocol consider peak angular velocities varying from a minimum of 3°/s to a maximum of about 200°/s, results in [17] are obtained using a different metric for accuracy (difference between range of motions) than geodesic paths in the quaternion space and thus a direct comparison is not possible.

A significant difference in performance was observed when passing from performing rotation against the gravity to rotations along the gravity axis. In the latter case, both sensor fusion algorithm heavily rely on magnetic field measurements in order to limit orientation drifts. When the magnetic field is locally perturbed (which is likely to be the case in indoor settings [28]), either wrong measurement are introduced in the sensor fusion framework or, in case a perturbation is detected, no reference measure at all is available to stabilize drifts on the heading component of the orientation. In both scenarios, the error affecting the orientation measure will increase. The same effect was also observed in the study from Cutti [13] and Bergamini [19], where higher angular errors were experienced for the IMU that was mainly exposed to rotations along the gravity vector during the experimental session. We also found that, when considering relative orientation among IMU, level of error to be expected is increased. This result is in agreement with the discussion in [16] and is motivated by the fact that each IMU tends to sense the reference geo-magnetical and gravity fields in a slightly different way, due to deviations in the sensor’s calibration parameters. Also, following the discussion by the same authors, the aforementioned effect can be rather mitigated than eliminated by an initial IMUs’ reference frame alignment. The results from the dynamic trial showed that, independently from the fusion algorithm used, the dynamics of the movements do have an effect on the performance of the tracker with both the algorithms experimented and as a general rule of thumb, the more the bandwidth and the amplitude, the more the error to be expected. Further, from the graphs in Figs 4 and 5 the dependency on the amplitude value results more evident for the *Heading* scenario.

With respect to the set of amplitudes considered in the trial, the choice was constrained by the capabilities of the robotic platform and therefore it is not representative of the full human range, e.g. amplitudes greater than 60°/ are easily achieved during human gait [29]. Despite the limitations of our testing setup, we do not expect a significant decrease in performance than the boundaries established in this study for those scenarios in which the orientation is mainly varied against gravity, e.g. that is the case of an IMU attached to the thigh for gait analysis.

The effect of time on accuracy investigated in [18] was not explicitly addressed as part of this study. Nonetheless, the analysis by Lebel and colleagues highlights that robustness against magnetic field variation is the major criticality to the use of IMU as orientation sensor, which is in agreement with the results reported in this study, i.e. the increased error in the heading tracking trial. With reference to the performance of the two sensor fusion algorithms used, the KF proved better in dynamic trials while the CF achieved a better static accuracy and with a faster convergence rate, though the numerical values of the error in both cases are still comparable. Moreover, the accuracy results presented are dependent on the choice of the tuning parameters for the sensor fusion algorithms. When a commercial system is used, this choice can still be possible in the form of a scenario selection, or in the version of the firmware that runs on the IMU device. Particularly, as highlighted by the results any improvements on the heuristics used for detecting and compensating magnetic distortion can have a significant impact on the boundaries of the errors reported.

Concluding, IMU based human motion measuring proves to be a valuable alternative to standard tools (e.g. stereophotogrammetry), as the greater portability and flexibility is traded off by a worse but limited level of errors (within 10° for the experimented scenarios). Nonetheless, the maturity of the technology and the algorithms for human applications is still poor. In fact, great expertise is required from the experimenter in order to properly use an IMU based systems, to identify the sources of error, e.g. starting from using a proper metric for quantifying the error itself, and to elaborate strategies to limit their effect, e.g. by implementing magnetic perturbation compensation algorithms [2] or domain specific assumptions [10].

## Supporting Information

S1 Table Dynamic accuracy table. Absolute and relative accuracy: numerical values of absolute and relative errors are reported as the median and the 95% UB, for all the experimental conditions. Reported values are expressed in °.
(PDF)

S1 Fig Magnetic field disturbances evaluation. The figure reports variation of the magnetic dip angle (top) and of the magnetic norm (bottom) expressed as median, interquartile range and 95% UB. The first bar represents data corresponding to no motion of the robot motors while the second are the data recorded during motor driving.
(PDF)
