# Supplementary material for: On the Orientation Error of IMU: Investigating Static and Dynamic Accuracy Targeting Human Motion
Source: PLoS One. 2016 Sep 9;11(9):e0161940. doi: 10.1371/journal.pone.0161940 (PMC5017605; doi:10.1371/journal.pone.0161940)
Supplement: S1 Table — Absolute and relative accuracy: numerical values of absolute and relative errors are reported as the median and the 95% UB, for all the experimental conditions. Reported values are expressed in °. (PDF) [file pone.0161940.s001.pdf]

S1 Table: **Dynamic accuracy Table.** All the reported values are in °. Accuracy is expressed as the **median value** : 95% range. AH. stands for Attitude and Heading movements.

ABSOLUTE ACCURACY

**Attitude**	**0.18 Hz**		**0.32 Hz**		**0.56 Hz**		**1.00 Hz**		**1.78 Hz**		**3.16 Hz**		**5.62 Hz**	
	KF	CF	KF	CF	KF	CF	KF	CF	KF	CF	KF	CF	KF	CF
**±3°**	**0.4**:1.0	**0.3**:0.9	**0.5**:1.8	**0.3**:1.0	**0.5**:1.5	**0.3**:1.0	**0.4**:1.5	**0.5**:1.5	**0.7**:2.0	**0.9**:2.0	**0.7**:2.0	**1.0**:3.9	**0.8**:2.5	**2.3**:5.7
**±6°**	**0.5**:1.6	**0.4**:1.3	**0.4**:1.0	**0.4**:1.4	**0.6**:1.9	**0.6**:2.3	**0.7**:1.5	**1.3**:3.7	**0.7**:1.8	**1.3**:3.1	**0.7**:1.8	**1.8**:3.5	**1.0**:3.5	**2.0**:3.2
**±5°**	**0.5**:2.0	**0.3**:0.7	**0.5**:1.7	**0.4**:1.2	**0.5**:1.9	**0.5**:2.0	**0.5**:2.0	**0.9**:2.0	**0.8**:2.3	**1.1**:2.4	**0.8**:2.3	**1.7**:4.3	–	
**±10°**	**0.5**:1.7	**0.5**:1.6	**0.5**:1.8	**0.7**:2.4	**0.5**:2.1	**1.0**:2.8	**0.7**:1.9	**1.6**:4.0	**0.9**:2.6	**2.1**:4.2	**0.8**:2.6	**3.0**:6.4	–	
**±9°**	**0.6**:1.7	**0.4**:1.4	**0.5**:1.9	**0.5**:2.2	**0.6**:2.0	**0.7**:1.7	**0.7**:2.3	**1.6**:4.3	**0.9**:2.4	**1.8**:3.8	–		–	
**±18°**	**0.6**:2.1	**0.8**:2.7	**0.6**:2.1	**0.8**:2.7	**0.7**:1.9	**1.7**:3.6	**0.8**:2.3	**2.2**:3.7	**0.9**:2.5	**3.3**:8.3	–		–	

**AH.**	**0.18 Hz**		**0.32 Hz**		**0.56 Hz**		**1.00 Hz**		**1.78 Hz**		**3.16 Hz**		**5.62 Hz**	
	KF	CF	KF	CF	KF	CF	KF	CF	KF	CF	KF	CF	KF	CF
**±3°**	**0.6**:2.3	**0.4**:1.2	**0.5**:3.0	**0.5**:1.6	**0.5**:2.6	**0.5**:1.6	**0.6**:2.0	**0.9**:2.1	**0.8**:2.7	**1.2**:2.2	**0.9**:3.0	**1.4**:4.2	**0.9**:2.8	**2.4**:4.9
**±6°**	**0.5**:2.1	**0.7**:2.4	**0.6**:2.4	**0.7**:2.4	**0.6**:2.3	**0.8**:2.5	**0.8**:2.7	**1.5**:2.8	**1.0**:3.1	**1.4**:2.7	**0.9**:2.6	**1.8**:4.6	**0.8**:2.9	**4.5**:8.2
**±5°**	**0.6**:3.0	**0.5**:2.1	**0.8**:3.4	**0.6**:2.0	**0.6**:2.3	**0.9**:2.1	**0.7**:2.2	**1.5**:3.0	**0.9**:2.9	**1.6**:3.2	**0.9**:2.8	**1.7**:5.1	–	
**±10°**	**0.7**:2.6	**1.1**:3.3	**0.7**:2.8	**1.3**:3.5	**0.6**:2.7	**1.5**:3.4	**0.7**:3.0	**1.8**:4.3	**0.9**:3.1	**1.8**:3.8	**1.0**:3.3	**3.2**:5.3	–	
**±9°**	**0.9**:4.4	**0.8**:3.1	**0.8**:2.9	**1.2**:3.1	**0.6**:2.7	**1.5**:3.9	**0.9**:3.0	**1.8**:4.0	**0.9**:3.0	**2.1**:4.0	–		–	
**±18°**	**0.6**:2.6	**1.6**:5.2	**0.8**:2.9	**1.9**:4.4	**1.0**:3.1	**2.4**:4.7	**1.0**:3.0	**2.1**:4.8	**1.1**:4.3	**3.1**:7.0	–		–	

**Heading**	**0.18 Hz**		**0.32 Hz**		**0.56 Hz**		**1.00 Hz**		**1.78 Hz**		**3.16 Hz**		**5.62 Hz**	
	KF	CF	KF	CF	KF	CF	KF	CF	KF	CF	KF	CF	KF	CF
**±3°**	**0.9**:2.7	**0.6**:2.7	**0.6**:2.8	**0.6**:2.4	**0.6**:2.2	**0.7**:2.2	**0.6**:1.9	**1.0**:2.8	**0.7**:2.3	**1.2**:2.5	**0.7**:2.1	**1.4**:2.7	**0.8**:1.9	**1.3**:4.4
**±6°**	**0.6**:2.0	**1.0**:4.7	**0.6**:1.9	**1.1**:3.8	**0.8**:2.6	**1.7**:3.4	**0.6**:1.9	**1.9**:4.2	**0.6**:2.2	**1.7**:3.6	**0.6**:1.9	**1.9**:4.2	**0.7**:2.1	**3.5**:4.9
**±5°**	**0.6**:1.9	**0.9**:2.5	**0.6**:2.6	**0.6**:2.6	**0.5**:2.2	**1.0**:3.7	**0.6**:2.3	**1.5**:2.9	**0.5**:1.9	**1.5**:2.6	**0.7**:2.2	**1.6**:4.0	–	
**±10°**	**0.5**:1.8	**1.1**:4.6	**0.8**:2.8	**0.9**:4.2	**0.6**:1.9	**1.6**:3.8	**1.1**:4.1	**2.2**:4.9	**0.7**:2.6	**1.7**:3.2	**1.4**:6.5	**3.0**:4.0	–	
**±9°**	**0.9**:3.3	**1.1**:4.1	**0.7**:3.0	**1.0**:4.0	**1.5**:4.2	**1.6**:4.3	**1.1**:3.3	**2.0**:3.3	**0.8**:2.8	**1.6**:3.4	–		–	
**±18°**	**0.7**:2.9	**1.9**:6.4	**1.3**:3.9	**1.9**:6.1	**1.0**:3.4	**2.4**:5.1	**1.0**:2.9	**2.2**:3.9	**1.1**:4.0	**2.8**:5.4	–		–	

RELATIVE ACCURACY

**Attitude**	**0.18 Hz**		**0.32 Hz**		**0.56 Hz**		**1.00 Hz**		**1.78 Hz**		**3.16 Hz**		**5.62 Hz**	
	KF	CF	KF	CF	KF	CF	KF	CF	KF	CF	KF	CF	KF	CF
**±3°**	**0.4**:1.2	**0.4**:1.8	**0.5**:1.8	**0.3**:1.2	**0.5**:1.5	**0.4**:1.4	**0.6**:1.6	**0.8**:2.0	**0.7**:1.8	**1.1**:2.7	**0.7**:2.0	**1.2**:3.1	**0.9**:2.4	**2.7**:5.6
**±6°**	**0.5**:1.4	**0.5**:1.5	**0.5**:1.4	**0.6**:1.7	**0.6**:2.2	**0.8**:2.4	**0.6**:1.3	**1.7**:3.4	**0.8**:1.7	**1.6**:4.7	**0.8**:1.8	**1.9**:5.0	**0.9**:2.6	**1.9**:4.5
**±5°**	**0.7**:2.2	**0.4**:1.0	**0.6**:1.9	**0.6**:2.0	**0.6**:1.7	**0.7**:2.3	**0.7**:2.0	**1.3**:2.7	**0.8**:2.1	**1.3**:3.4	**0.8**:1.9	**2.1**:3.8	–	
**±10°**	**0.6**:1.4	**0.6**:2.0	**0.6**:1.6	**1.0**:2.9	**0.7**:2.0	**1.3**:2.8	**0.8**:1.9	**2.0**:3.9	**1.0**:2.3	**1.9**:3.7	**0.9**:2.4	**3.5**:6.9	–	
**±9°**	**0.7**:2.0	**0.6**:2.2	**0.6**:1.7	**0.8**:2.5	**0.6**:1.7	**1.0**:1.9	**0.8**:2.2	**2.1**:4.9	**0.8**:2.2	**2.1**:3.8	–		–	
**±18°**	**0.7**:1.7	**1.1**:3.0	**0.8**:2.2	**1.1**:3.0	**0.8**:1.6	**1.8**:3.3	**0.9**:2.0	**2.2**:4.4	**0.9**:2.3	**3.2**:6.9	–		–	

**AH.**	**0.18 Hz**		**0.32 Hz**		**0.56 Hz**		**1.00 Hz**		**1.78 Hz**		**3.16 Hz**		**5.62 Hz**	
	KF	CF	KF	CF	KF	CF	KF	CF	KF	CF	KF	CF	KF	CF
**±3°**	**0.6**:2.2	**0.5**:1.8	**0.8**:4.2	**0.6**:2.2	**0.9**:3.8	**0.6**:2.1	**0.8**:2.9	**1.1**:2.6	**1.0**:3.4	**1.3**:2.8	**1.1**:3.4	**1.6**:5.0	**1.1**:3.3	**1.8**:3.6
**±6°**	**0.7**:2.5	**1.0**:2.6	**0.9**:2.6	**0.9**:2.7	**0.7**:2.9	**1.0**:2.3	**1.1**:3.3	**1.6**:3.3	**1.3**:4.4	**1.8**:3.6	**1.1**:3.2	**2.1**:6.8	**1.0**:3.1	**1.2**:4.5
**±5°**	**0.8**:3.2	**0.8**:3.9	**0.9**:3.7	**0.9**:2.9	**0.8**:2.8	**1.1**:2.4	**0.9**:3.4	**1.7**:3.8	**1.3**:3.4	**1.9**:3.9	**1.0**:3.2	**2.0**:4.5	–	
**±10°**	**0.9**:2.7	**1.3**:4.6	**0.9**:3.5	**1.4**:4.4	**0.9**:4.0	**1.9**:4.2	**1.0**:4.3	**2.2**:4.3	**1.1**:4.0	**2.5**:4.4	**1.3**:3.9	**2.7**:7.2	–	
**±9°**	**1.0**:4.4	**1.0**:3.7	**1.0**:3.5	**1.3**:3.5	**0.8**:3.1	**2.1**:4.4	**1.1**:3.5	**1.9**:4.2	**1.1**:4.1	**2.2**:4.6	–		–	
**±18°**	**0.9**:3.1	**1.5**:7.1	**1.1**:4.2	**1.7**:6.0	**1.1**:3.8	**2.4**:5.9	**1.3**:3.8	**2.3**:4.5	**1.3**:6.5	**3.5**:8.4	–		–	

**Heading**	**0.18 Hz**		**0.32 Hz**		**0.56 Hz**		**1.00 Hz**		**1.78 Hz**		**3.16 Hz**		**5.62 Hz**	
	KF	CF	KF	CF	KF	CF	KF	CF	KF	CF	KF	CF	KF	CF
**±3°**	**0.7**:2.2	**0.8**:3.9	**1.0**:5.7	**0.6**:3.6	**0.9**:3.5	**0.8**:3.4	**0.9**:3.1	**1.2**:4.2	**1.1**:4.4	**1.1**:3.2	**1.0**:3.2	**1.3**:3.6	**1.0**:3.0	**1.8**:5.2
**±6°**	**1.0**:3.3	**1.0**:5.1	**0.9**:3.5	**1.0**:6.3	**1.2**:5.0	**1.4**:4.9	**1.1**:3.7	**2.0**:5.3	**1.1**:3.6	**1.8**:5.0	**1.0**:3.6	**2.2**:4.9	**1.3**:4.6	**2.1**:6.1
**±5°**	**0.7**:2.5	**0.6**:2.7	**0.9**:4.6	**0.7**:2.7	**1.2**:4.2	**1.1**:3.9	**1.3**:5.4	**1.3**:2.8	**1.2**:4.5	**1.3**:3.3	**1.1**:4.5	**2.0**:4.1	–	
**±10°**	**1.0**:5.1	**0.7**:4.1	**1.7**:6.0	**0.9**:4.2	**1.4**:4.9	**1.4**:4.5	**2.3**:8.2	**1.7**:4.5	**1.6**:6.0	**2.0**:3.9	**2.7**:10.3	**2.0**:3.6	–	
**±9°**	**1.4**:6.4	**0.9**:4.4	**1.3**:5.1	**0.9**:3.6	**2.1**:7.3	**1.9**:5.1	**1.8**:6.3	**1.5**:3.9	**1.5**:5.5	**1.5**:4.2	–		–	
**±18°**	**1.7**:5.2	**1.3**:6.9	**2.3**:9.4	**2.2**:6.1	**1.6**:7.8	**2.4**:5.5	**1.9**:6.9	**2.4**:4.3	**2.2**:9.4	**3.1**:6.3	–		–	
