# Supplementary material for: On the Orientation Error of IMU: Investigating Static and Dynamic Accuracy Targeting Human Motion
Source: PLoS One. 2016 Sep 9;11(9):e0161940. doi: 10.1371/journal.pone.0161940 (PMC5017605; doi:10.1371/journal.pone.0161940)
Supplement: S1 Fig — The figure reports variation of the magnetic dip angle (top) and of the magnetic norm (bottom) expressed as median, interquartile range and 95% UB. The first bar represents data corresponding to no motion of the robot motors while the second are the data recorded during motor driving. (PDF) [file pone.0161940.s002.pdf]

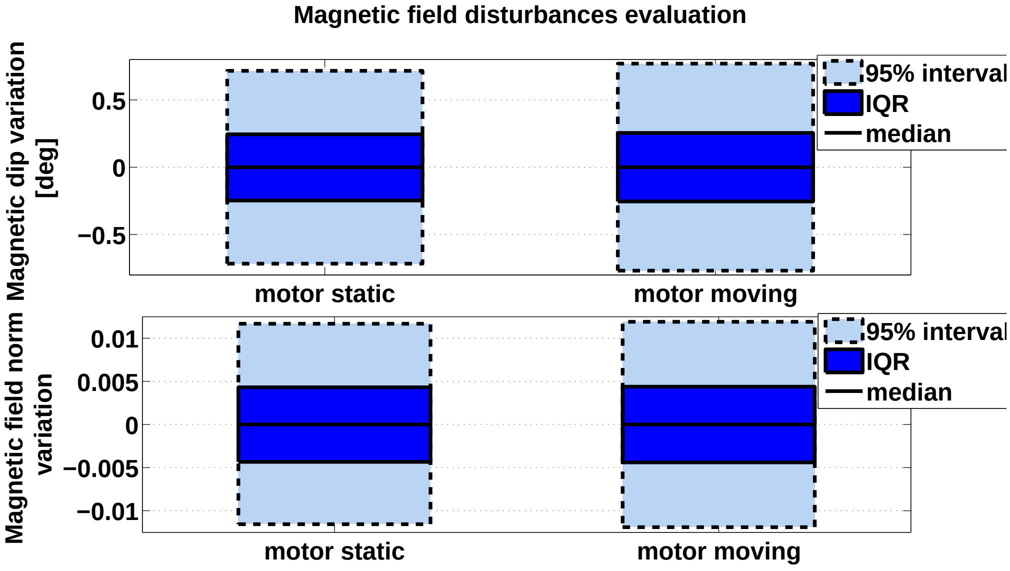

S1 Fig. Magnetic field disturbances evaluation. The figure reports variation of the magnetic dip angle (top) and of the magnetic norm (bottom) expressed as median, interquartile range and 95% UB. The first bar represents data corresponding to no motion of the robot motors while the second are the data recorded during motor driving. These data refer to a preliminary experiment aimed at evaluating the effect of using the KUKA LWR 4+ robotic manipulator on the magnetic field sensed by IMU's magnetometers. The robot's housing is made of aluminum, stainless steel (paramagnetic materials) and ABS plastic. Therefore, the only active source of magnetic field disturbance related to the use of the robotic arm is due to permanent magnets in motors and electro–magnetic waves generated by motor driving. In order to isolate those effects on the proposed sensors setup, we set the robot to a fully extended configuration, namely "candle" configuration. Then, we drove the pair of aligned robot joints E1 and A6 with a counterphase sinusoidal input. By doing so, the IMUs attached to the robot EE are kept static while motors are instead in motion and perturbing nearby magnetic field. Particularly, each of the involved joint was driven with a set of sinusoidal input, sharing the same amplitude and frequency but with a 180° phase shifting. The perturbation on the magnetic field was evaluated in terms of its effect on the variation both of the ℓ^2–norm of the magnetic field and of the magnetic dip angle. The latter depends on the position on Earth's surface (between 60 and 70 degrees at our latitude) and can be computed from the accelerometer and magnetometer measurements as: $\theta_{dip} = \frac{\pi}{2} - cos^{-1}(\frac{\mathbf{g}_B \cdot \mathbf{m}_B}{\|g\|_2 \|m\|_2})$, where $\mathbf{g}_B$ and $\mathbf{m}_B$ are the gravity vector (measured by accelerometer in static condition) and the magnetic flux vector in sensor body frame (B). In fact, the variation of this quantities in time (defined as: $\Delta_{\theta_{dip}}(t) = \theta_{dip}(t) - \theta_{dip}(t - T_s)$, $\Delta_{\|m\|_2}(t) = \|\mathbf{m}_B\|_2(t) - \|\mathbf{m}_B\|_2(t - T_s)$, where T_s is the IMU sampling time) is the input to magnetic field disturbances compensation strategies that are typically integrated in the orientation estimation framework of an IMU. Experimental results for a 40 s motor static period followed by a 40 s counterphase motor moving (60° peak–to–peak amplitude at 0.5 Hz) are reported the figure. A slight increase in the IQR for the dip angle variation (+3%) and the magnetic field norm variation (+1%) is observed. Since distribution of $\Delta_{\theta_{dip}}$ and $\Delta_{\|m\|_2}$ is not Gaussian, we used Wilkoxon rank–sum test for the analysis of results. No statistical difference between the "motor static" and the "motor moving" groups is observed with a 5% significance level ($p > 0.95$).
